# Elevated levels of interleukin‐33 are associated with asthma: A meta‐analysis

**DOI:** 10.1002/iid3.842

**Published:** 2023-04-19

**Authors:** Ranran Zhao, Yun Shi, Na Liu, Bin Li

**Affiliations:** ^1^ Department of Respiratory Medicine Capital Medical University Affiliated Beijing Friendship Hospital Beijing China; ^2^ Medical and Health Center Capital Medical University Affiliated Beijing Friendship Hospital Beijing China; ^3^ Department of Respiratory Medicine Beijing Hepingli hospital Beijing China

**Keywords:** asthma, interleukin‐33, meta‐analysis, serum, systematic review

## Abstract

**Background:**

Previous studies reported that patients with asthma showed higher levels of interleukin (IL)‐33 in peripheral blood, compared to healthy control (HCs). However, we also noticed that there were no significant differences of IL‐33 levels between controls and asthma patients in a recent study. We aim to conduct this meta‐analysis and evaluate the feasibility of IL‐33 in peripheral blood that may act as a promising biomarker in asthma.

**Methods:**

Articles published before December 2022 were searched in these databases (PubMed, Web of Science, EMBASE, and Google Scholar). We used STATA 12.0 software to compute the results.

**Results:**

The study showed that asthmatics showed higher IL‐33 level in serum and plasma, compared to HCs (serum: standard mean difference [SMD] 2.06, 95% confidence interval [CI] 1.12−3.00, *I* ^2^ = 98.4%, *p* < .001; plasma: SMD 3.67, 95% CI 2.32−5.03, *I* ^2^ = 86.0%, *p* < .001). Subgroup analysis indicated that asthma adults showed higher IL‐33 level in serum, compared to HCs, whereas no significant difference in IL‐33 level in serum was showed between asthma children and HCs (adults: SMD 2.17, 95% CI 1.09−3.25; children: SMD 1.81, 95% CI −0.11 to 3.74). The study indicated that moderate and severe asthmatics showed higher IL‐33 level in serum, compared to mild asthmatics (SMD 0.78, 95% CI 0.41−1.16, *I* ^2^ = 66.2%, *p* = .011).

**Conclusions:**

In conclusion, the main findings of present meta‐analysis suggested that there was a significant correlation between IL‐33 levels and the severity of asthma. Therefore, IL‐33 levels of either serum or plasma may be regarded as a useful biomarker of asthma or the degree of disease.

## Graphical abstract

In conclusion, the main findings of present meta‐analysis suggested that there was significant correlation between interleukin (IL)‐33 levels and the severity of asthma. Therefore, the IL‐33 levels of either serum or plasma may be regarded as a useful biomarker of asthma or the degree of disease. However, we can not ignore the influences of ethnic and age factors on the IL‐33 levels. It is encouraged that data from multi‐center, well‐designed and large sample size studies should be conducted for validating the clinical value of IL‐33 on asthma.

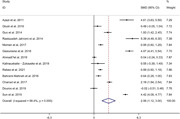

## 1 INTRODUCTION

Asthma is one of the most common chronic inflammatory diseases of the airways and affects more than 300 million patients globally.^1^ Asthma is usually associated with airway inflammation and hyperresponsiveness, but these are not necessary or sufficient for diagnosis.^2^ For both adults and children, asthma is a serious health problem which is characterized by recurrent wheezing and airflow obstruction.^3^ Epidemiological statistics report that the prevalence, severity and mortality of asthma differ around the world.^4^ Among children boys are more likely to suffer from asthma than girls, while asthma prevalence is higher in females patients than in male patients.^5^ Patients with severe asthma are at increased risk for substantial disability, impaired life quality and life‐threatening exacerbations.^6^

In recent years, an enormous amount of research has been directed toward finding novel biomarkers of asthma. Ideally, samples should be collected using quick, cost‐efficient and noninvasive methods. Thus, some serum/plasma biomarkers change in asthma have been searched in recent years. Interleukin‐4 (IL‐4), IL‐5, and IL‐13 are involved in asthma pathogenesis by synthesis of immunoglobulin E (IgE), activation of eosinophils, and by inducing hypersecretion of mucus resulting in hyperresponsiveness of airways.^7^ Periostin, a matricellular protein belonging to the fasciclin family, is a key molecule linking type‐2 airway inflammation and airway remodeling. Serum periostin is elevated in the eosinophilic/type 2 subtype of severe asthma.^8^ Dipeptidyl peptidase (DPP)‐4 is a serine protease involved in glucose metabolism and immune regulation via its cleavage of various growth factors, cytokines and chemokines.^9^ Serum DPP‐4 is generally similar in asthma patients compared to healthy controls (HCs), or lower in patients with more severe asthma.^10^, ^11^ Previous studies demonstrated that these serum/plasma biomarkers have thus‐far been disappointing in their clinical utility. Thus, exploring a new biomarker of asthma is essential for asthma diagnosis.

IL‐33 is a member of the IL‐1 cytokine family and abundantly expressed by fibroblasts, endothelial and epithelial cells.^12^ IL‐33, as an alarm signal, is released to alert immune cells which express the IL‐1 receptor like‐1 (IL‐1RL1 or ST2) when cells damage or tissues injury occur.^13^ Recent studies reported that IL‐33 involves in lots of physiological and pathophysiological actions including host defense, the immune signaling for intestinal homeostasis host and cardiac fibrosis.^14^, ^15^ Moreover, IL‐33 results in type 2 responses by activating allergic inflammation‐related eosinophils, basophils, mast cells, macrophages, and group 2 innate lymphoid cells (ILC2s) through IL‐1RL1.^16^, ^17^

Previous studies reported that patients with asthma showed higher levels of IL‐33 in peripheral blood, compared to HCs.^18^, ^19^ However, we also noticed that there were no significant differences of IL‐33 levels between controls and asthma patients in a recent study.^20^ We aim to conduct this meta‐analysis and evaluate the feasibility of IL‐33 in peripheral blood that may act as a promising biomarker in asthma.

## 2 METHODS

**Figure 1:**
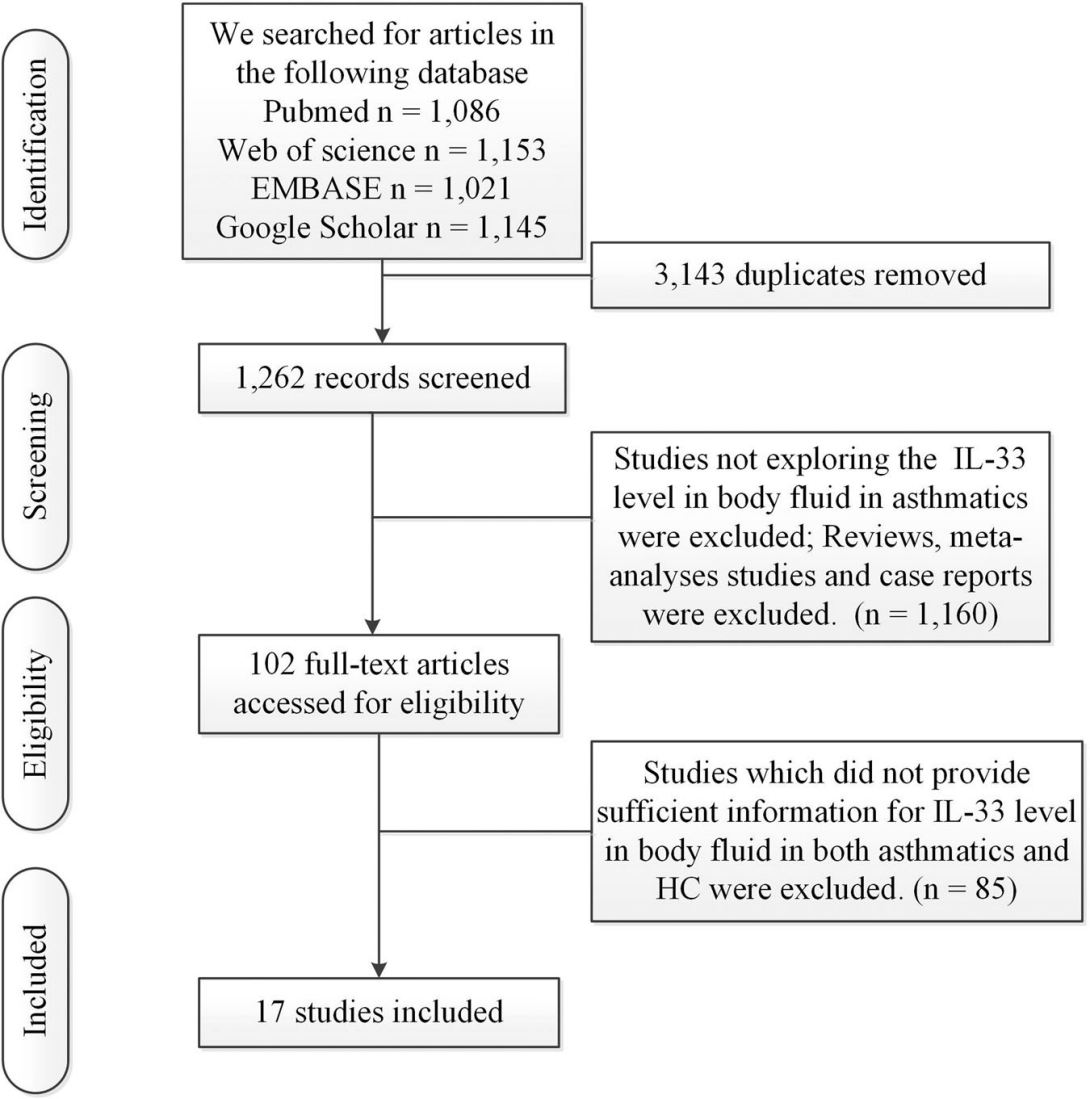
Flow of information through the meta‐analysis.

The study was made according to the Preferred Reporting Items for Systematic reviews and Meta‐Analysis (PRISMA) guideline.^21^ The PRISMA Checklist is included in the Supporting Information: Table 1. The process for selecting studies (including identification, screening, eligibility, included in the meta‐analysis) was showed in Figure 1.

### 2.1 Search strategy

Articles published before December 2022 were searched in these databases (PubMed, Web of Science, EMBASE and Google Scholar). These search terms were used: (“Interleukin‐33” OR “Interleukin 33” OR “IL33” OR “IL‐33”) AND (“asthma”). After excluding *N* = 3143 duplicates, *N* = 1262 studies were included in the study. Two independent investigators (Ranran Zhao and Yun Shi) decided study eligibility after reading the scanned abstracts and citations. We resolved discrepancies through consultation.

### 2.2 Inclusion and exclusion criteria

We included studies according to the following inclusion criteria: (1) studies explored IL‐33 in peripheral blood; (2) studies focused on asthma. Exclusion criteria were showed as follows: (1) reviews, meta‐analyses studies and case reports; (2) published articles written in languages other than English; (3) studies which did not provide sufficient information for IL‐33 level in the peripheral blood in both asthmatics and HC.

### 2.3 Data collection

The following data were collected from included articles using Excel by two independent investigators (Ranran Zhao and Yun Shi): author, publication year, located country, ethnicity, sample size, gender, mean age, specimen, detection method and results (comparison in IL‐33 level in peripheral blood between asthmatics and HC). If data of these items were not reported in primary studies, the item was marked as “not reported (NR).”

### 2.4 Meta‐analysis

We used STATA 12.0 software to compute the results. The SMD and a 95% confidence interval (CI) regarding comparison in IL‐33 level in the peripheral blood between asthmatics and HC were computed. Q test and *I* ^2^ were used to assess heterogeneity among studies. With low heterogeneity (*p* Value for Q test > .05 and *I* ^2^ < 50%), fixed effects models were used; with high heterogeneity (*p* Value for Q test ≤ .05 and *I* ^2^ ≥ 50%), random effects models were used. Meta‐regression analyses were made to evaluate the source of the heterogeneity. Subgroup analyses (for children or adult, different ethnicities, different severity) were made to investigate the source of the heterogeneity. In addition, sensitivity analysis was made by removing 1 individual study each time to assess the stability of the meta‐analysis. Publication bias was evaluated by Begg's test, Egger's test and funnel plots.

## 3 RESULTS

### 3.1 Study characteristics

Table 1 reported characteristics of 17 finally included studies. *N* = 13 studies^18^, ^19^, ^20^, ^22^, ^24^, ^25^, ^27^, ^28^, ^30^, ^31^, ^32^, ^33^, ^35^ (including 1026 asthmatics and 880 HCs) compared IL‐33 level in serum between asthmatics and HCs. *N* = 3 studies^26^, ^29^, ^34^ (including 364 asthmatics and 303 HCs) compared IL‐33 level in plasma between asthmatics and HCs. *N* = 4 studies^23^, ^30^, ^31^, ^32^ (including 189 moderate and severe asthmatics and 127 mild asthmatics) compared IL‐33 level in serum between moderate and severe asthmatics and mild asthmatics.

**Table 1:** Study characteristics.

Study	Country	Sample size (case/control)	Male (case/control)	Mean age (case/control)	Specimen	Detection	Result (pg/ml)
Azazi et al.^22^	Egypt	30/30	16/15	36.7/34.5	serum	ELISA (WKEA MED)	Case versus control: 846,000 ± 202,150/174,000 ± 41,200
Hamzaoui et al.^23^	Tunisia	37/22	25/12	9.8/6.5	Serum and induced sputum	ELISA (GenWay Biotech)	Case versus control: sputum: 1600 ± 500/860 ± 90
							Moderate versus mild asthma: serum: 1870 ± 710/1120 ± 290 sputum: 1960 ± 390/1200 ± 230
Guo et al.^24^	China	45/40	24/23	39.56/43.55	serum	ELISA (R&D Systems)	Case versus control: 903.62 ± 523.78/158.1 ± 81.74
Raeiszadeh Jahromi et al.^25^	India	44/44	22/22	NR/38.4	serum	ELISA (Ray Biotech)	Case versus control: 79.10 ± 20.62/0.51 ± 0.26
Chauhan et al.^26^	India	65/15	52/11	6.4/8.0	plasma	ELISA (Ray Biotech)	Case versus control: 4404 ± 413/3282 ± 331.5
Bahrami Mahneh et al.^27^	Iran	61/63	38/30	9.44/9.97	serum	ELISA (eBiosience)	Case versus control: 15.17 ± 32.3/0.61 ± 2.16
Gluck et al. ^28^	Poland	44/19	8/8	43/44	serum and exhaled breath condensate (EBC)	ELISA (R&D Systems)	Case versus control: serum: 58.8 (36−157)/31 (23−47) EBC: 5 (2.2−9)/1.9 (1.2−2.6)
Rosser et al.^29^	USA	294/283	170/136	10.1/10.5	plasma	Bio‐Plex HTF system (Bio‐Rad Laboratories)	Case versus control: 57.54 ± 3.72/38.90 ± 6.03
Charrad et al.^30^	Tunisia	100/100	65/65	9.1/9.5	serum	ELISA (GenWay Biotech)	Case versus control: 1.48 ± 0.47/0.70 ± 0.18
							Moderate and severe versus mild asthma: 1.71 ± 0.54/1.24 ± 0.17
Momen et al. ^31^	Iran	90/57	40/23	41/41	serum	ELISA (BioLegend)	Case versus control: 322.6 ± 241/139.7 ± 68.17
							Severe versus moderate versus mild asthma: 704.7 (615.1−794.2)/380.4 (325.3−435.5)/202.6 (182.1−223.2)
Gasiuniene et al. ^32^	Lithuania	115/85	35/33	45.82/44.91	serum	ELISA (BioLegend)	Case versus control: 672.73 ± 104.47/268.52 ± 27.56
							Severe versus moderate versus mild asthma: 684.82 ± 221.12/603.07 ± 138.48/574.39 ± 201.54
Ahmadi et al.^33^	Iran	140/72	47/33	41.8/35.3	serum	ELISA (Boster)	Case versus control: 3767.5 ± 1139.8/3712.8 ± 1418.3
Bhowmik et al. ^34^	India	5/5	2/3	62/34	plasma	ELISA (RayBio)	Case versus control: 9.033 ± 0.585/2.35 ± 0.308
Douros et al. ^20^	Greece	44/26	26/17	8.9/8.6	serum	ELISA (Boster Biological Technology)	Case versus control: 313.7 ± 324.2/320.5 ± 310.2
Kalinauskaite‐Zukauske et al. ^35^	Lithuania	9/9	5/1	25/30	serum	ELISA (R&D Systems)	Case versus control: 65.7 (28.6−119.3)/37.8 (13.0−19.9)
Sun et al.^19^	China	204/235	113/137	7.5/8.5	serum	ELISA (R&D Systems)	Case versus control: 127.82 ± 15.79/73.30 ± 8.20
Rabea et al.^18^	Egypt	100/100	35/40	45.5/54.5	serum	ELISA (PicoKine)	Case versus control: 45 (39.8−57)/17 (14−19.8)

Abbreviations: ELISA, enzyme linked immunosorbent assay; NR, not reported; USA, United States.

### 3.2 Meta‐analysis results

**Figure 2:**
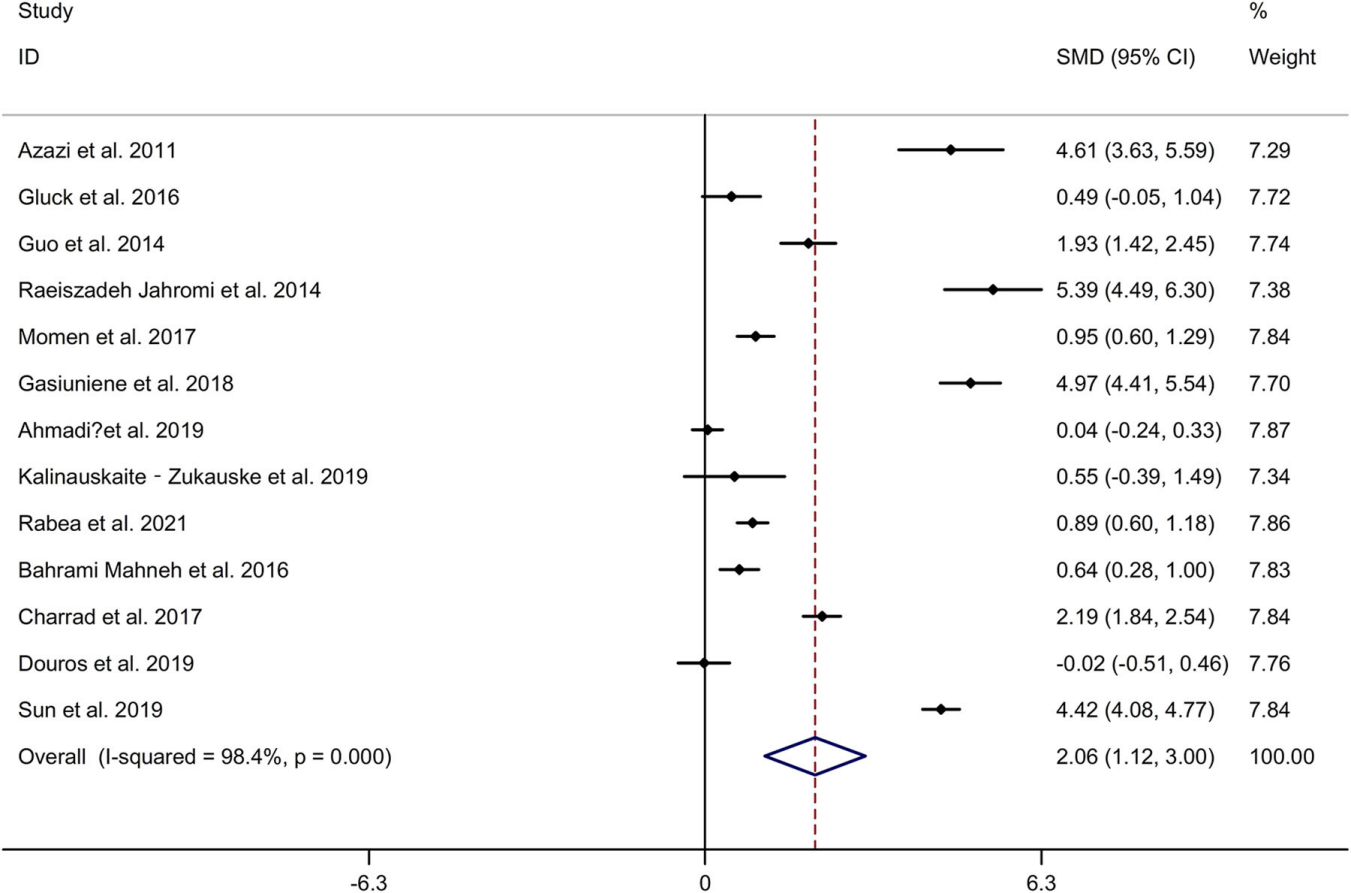
Forest plots regarding comparison in IL‐33 level in serum between asthmatics and HCs. CI, confidence interval; HC, healthy control; IL, interleukin; SMD, standard mean difference.

**Figure 3:**
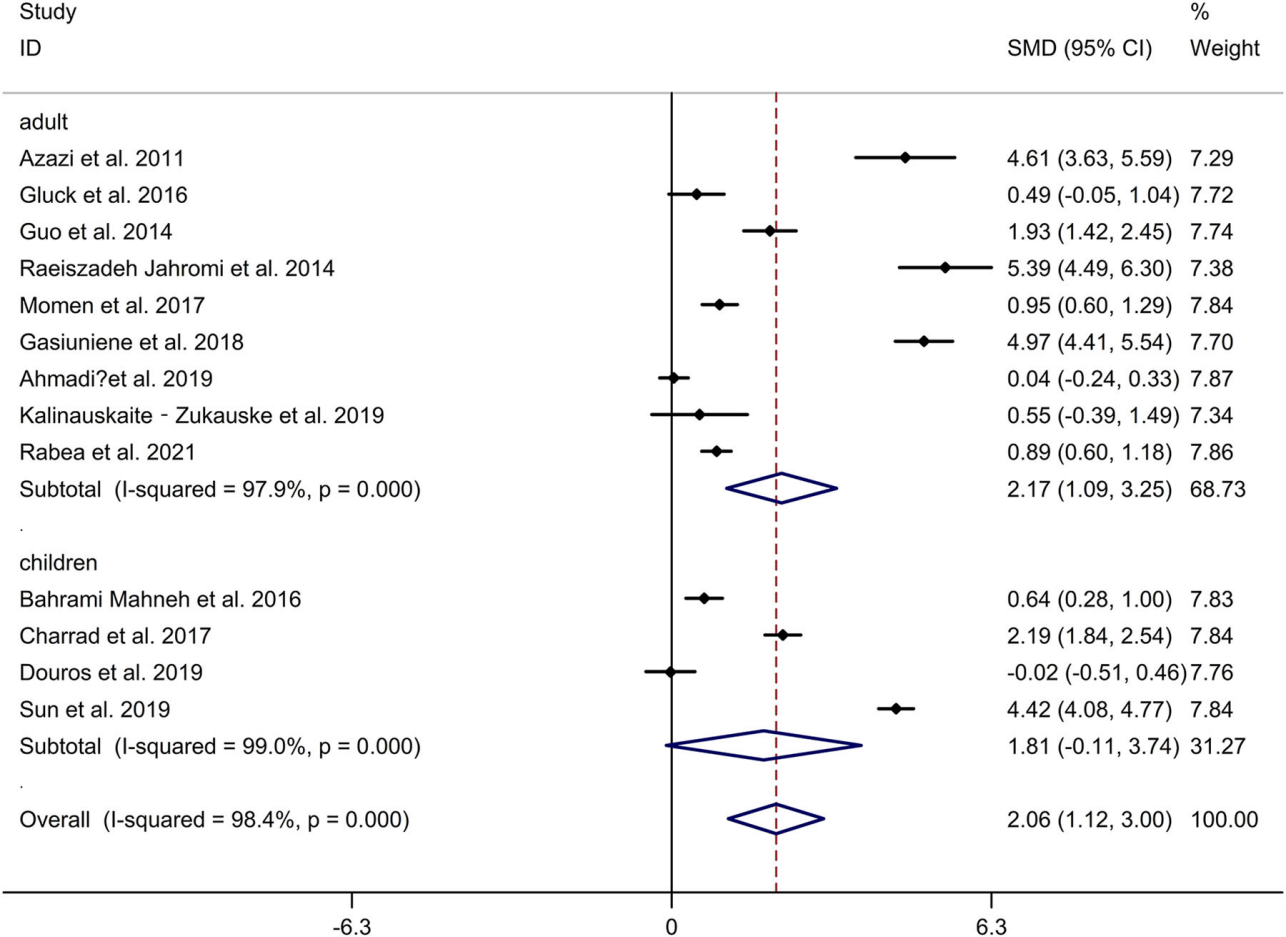
Subgroup analysis regarding comparison in IL‐33 level in serum between asthmatics and HCs in asthma adults and asthma children. CI, confidence interval; HC, healthy control; IL, interleukin; SMD, standard mean difference.

The study showed that asthmatics showed higher IL‐33 level in serum, compared to HCs (SMD 2.06, 95% CI 1.12−3.00, *I* ^2^ = 98.4%, *p* < .001; Figure 2). Meta‐regression analysis showed that publication year and gender were not responsible for heterogeneity across studies (publication year: *p* = .152; gender: *p* = .987). *N* = 9 studies^18^, ^22^, ^24^, ^25^, ^28^, ^31^, ^32^, ^33^, ^35^ (including 617 asthma adults and 456 HCs) compared IL‐33 level in serum between asthma adults and HCs. *N* = 4 studies^19^, ^20^, ^27^, ^30^ (including 409 asthma children and 424 HCs) compared IL‐33 level in serum between asthma children and HCs. Subgroup analysis indicated that asthma adults showed higher IL‐33 level in serum, compared to HCs, whereas no significant difference in IL‐33 level in serum was showed between asthma children and HCs (adults: SMD 2.17, 95% CI 1.09−3.25; children: SMD 1.81, 95% CI −0.11 to 3.74; Figure 3). Subgroup analysis indicated that asthmatics showed higher IL‐33 level in serum in Asian, compared to HCs, whereas no significant difference in IL‐33 level in serum was showed between asthmatics and HCs in Caucasian (Asian: SMD 2.21, 95% CI 0.64−3.77; Caucasian: SMD 1.65, 95% CI −0.05 to 3.34; Supporting Information: Figure 1). Sensitivity analyses showed no changes in the direction of effect while anyone study was excluded for meta‐analysis (Supporting Information: Figure 2). Begg's test, Egger's test and funnel plot reported no significant risk of publication bias (Begg's test: *p* = .180; Egger's tests: *p* = .216; Supporting Information: Figure 3).

**Figure 4:**
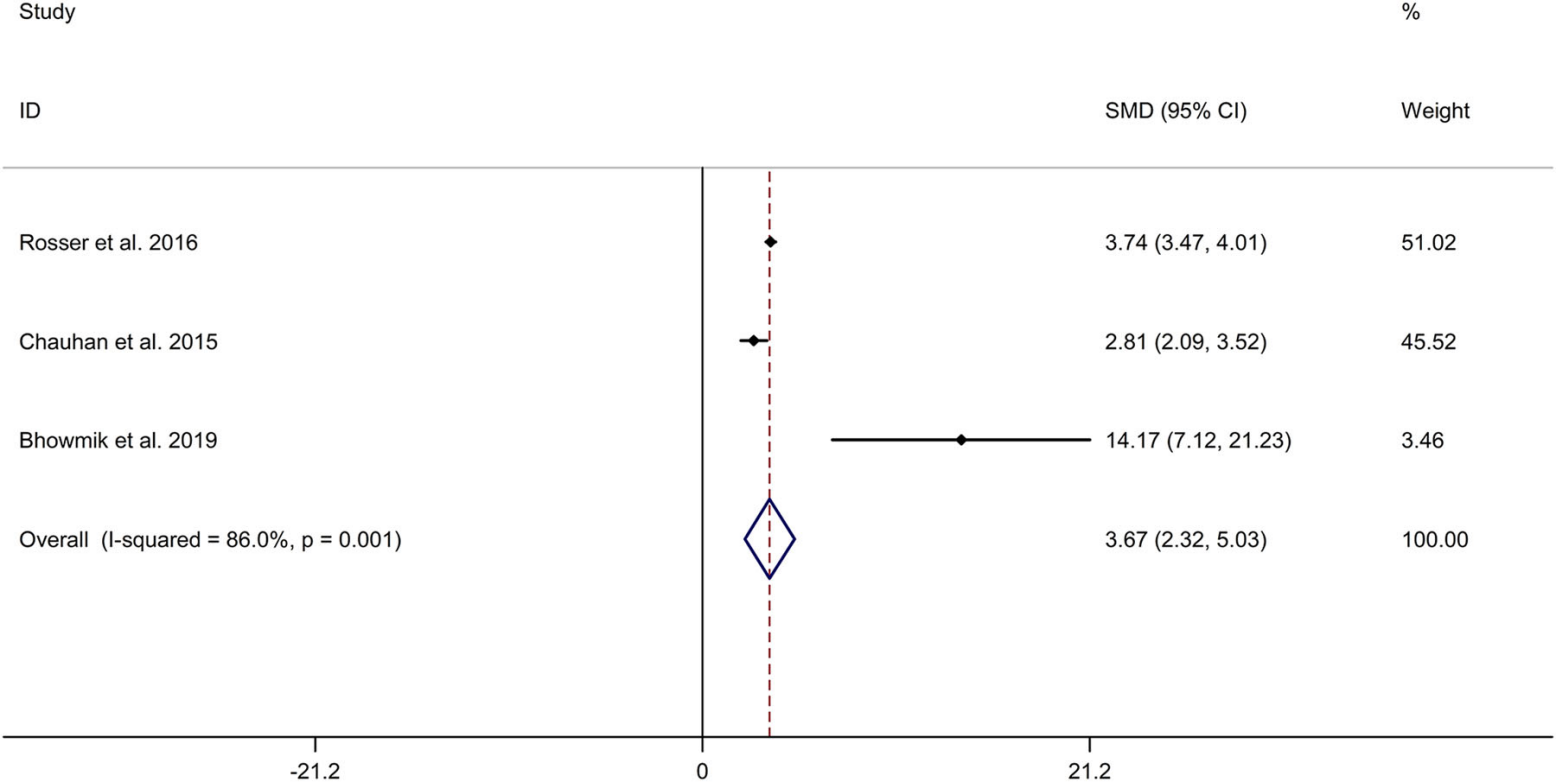
Forest plots regarding comparison in IL‐33 level in plasma between asthmatics and HCs. CI, confidence interval; HC, healthy control; IL, interleukin; SMD, standard mean difference.

The meta‐analysis showed that asthmatics showed higher IL‐33 level in plasma, compared to HCs (SMD 3.67, 95% CI 2.32−5.03, *I* ^2^ = 86.0%, *p* < .001; Figure 4). Meta‐regression analysis showed that publication year was not responsible for heterogeneity across studies (*p* = .232). Sensitivity analyses showed no changes in the direction of effect while anyone study was excluded for meta‐analysis (Supporting Information: Figure 4). Begg's test, Egger's test and funnel plot reported no significant risk of publication bias (Begg's test: *p* = .602; Egger's tests: *p* = .783; Supporting Information: Figure 5).

**Figure 5:**
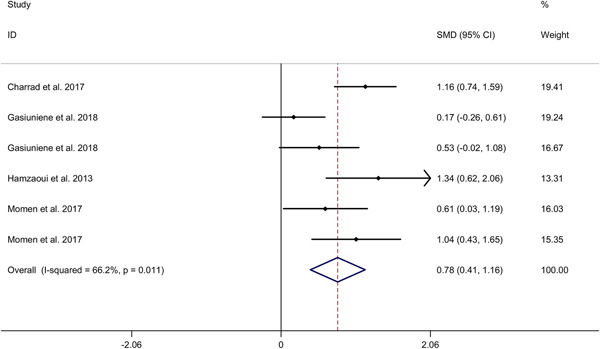
Forest plots regarding comparison in IL‐33 level in serum between moderate/severe asthmatics versus mild asthmatics. CI, confidence interval; HC, healthy control; IL, interleukin; SMD, standard mean difference.

The study indicated that moderate and severe asthmatics showed higher IL‐33 level in serum, compared to mild asthmatics (SMD 0.78, 95% CI 0.41−1.16, *I* ^2^ = 66.2%, *p* = .011; Figure 5). Meta‐regression analysis showed that publication year was not responsible for heterogeneity across studies (*p* = .134). Subgroup analysis indicated that moderate asthmatics showed higher IL‐33 level in serum, compared to mild asthmatics (SMD 0.66, 95% CI 0.02 to 1.30; Supporting Information: Figure 6). Subgroup analysis for different ethnicities could not be obtained because of the limited number of included studies (Supporting Information: Figure 7). Sensitivity analyses showed no changes in the direction of effect while anyone study was excluded for meta‐analysis (Supporting Information: Figure 8). Begg's test, Egger's test and funnel plot reported no significant risk of publication bias (Begg's test: *p* = .348; Egger's tests: *p* = .505; Supporting Information: Figure 9).

## 4 DISCUSSION

In this meta‐analysis, we included 17 studies with a total of over 2600 participants (1427 asthma patients and 1205 HCs). Our findings showed that asthma patients were likely to have higher serum levels of IL‐33, compared to HCs (SMD = 2.06, 95% CI: 1.12−3.00). This result was analogous to previous meta‐analysis conducted by Li et al.^36^ (*p* = .02, 95% CI: 7.57 to 72.74). Subgroup analysis revealed that the difference of serum IL‐33 existed among adults, while not among children (adults: SMD = 2.17, 95% CI: 1.09−3.25; children: SMD = 1.81, 95% CI: −0.11 to 3.74). However, Wang et al. reported that there was an association between asthma patients and higher serum levels of IL‐33 among children (SMD = 1.32, 95% CI: 0.54−2.10).^37^ The differences in results may be due to the inclusion of non‐English literatures. Wang et al. included 4 articles written in languages rather than English. Moreover, we found that the racial difference may influence the results (Asian: SMD = 2.21, 95% CI: 0.64−3.77; Caucasian: SMD = 1.65, 95% CI: −0.05 to 3.34). We found that the result of plasma was similar to that of serum (plasma: SMD = 3.67, 95% CI: 2.32−5.03). Regarding the impact of disease duration on serum IL‐33, no study reports the duration at which IL‐33 remains elevated in the serum. Momen et al.^31^ reported no significant association between serum IL‐33 level and disease duration. Raeiszadeh et al.^25^ found no difference in serum IL‐33 level between patients with ≤5 years asthma duration and those with >5 years asthma duration. More longitudinal studies are essential to explore the duration at which IL‐33 remains elevated in the serum. Some studies explored the association between IL‑33 in peripheral blood and some other biomarkers (including total IgE and sputum eosinophils) in asthma. Momen et al.^31^ observed a significant direct association of IL‑33 with total IgE level in the asthmatic group. However, Gasiuniene et al.^32^ reported that no significant correlation was found between IL‐33 in serum and IgE levels. Guo et al.^24^ reported no significant correlation between serum level of IL‐33 and total IgE. In addition, Guo et al.^24^ reported that serum level of IL‐33 failed to correlate with sputum eosinophils percentage (EOS%). More studies are needed to explore the association between IL‐33 level in peripheral blood and those biomarkers currently in use.

Meanwhile, this meta‐analysis revealed that the serum levels of IL‐33 were higher in moderate and severe asthma patients compared to that in mild asthma patients (SMD = 0.78, 95% CI: 0.41−1.16). This result was inconsistent with finding from Wang et al.^37^ Wang et al. demonstrated that there were no significant differences of serum IL‐33 between severe versus moderate asthma (SMD = 0.35, 95% CI: −0.08 to 0.78) and moderate versus mild asthma (SMD = 0.69, 95% CI: ‐0.47 to 1.86) among children, respectively. It seems to be due to data of adult asthma in our study.

IL‐33 plays important roles in type 2 inflammatory responses and linking innate and adaptive immunity.^38^ Genome‐wide association study (GWAS) indicated that the gene polymorphisms of IL‐33/IL‐1R1 pathway showed association with development of wheeze and asthma in early childhood.^39^ As a key target of IL‐33 enriched in the lungs, ILC2s would proliferate significantly and secreted certain cytokines such as IL‐5 in response to the stimulation of IL‐33.^40^, ^41^ Sun et al. showed that CD146 may participate in the airway remodeling of chronic asthma via IL‐33 signaling pathway.^42^ Most notably, IL‐33 also plays a critical role in innate immunity in gut and the regulation of adipose thermogenesis.^43^, ^44^

There were some limitations in this study. First, we only included the published articles in English. The findings may be biased owing to the exclusion of unpublished and non‐English literatures. Second, several confounding factors were not adjusted for all included studies, including gender, family history, diet differences and cigarette smoking, which may influence the results. Thirdly, the study did not explore IL‐33 levels in the airways (induced sputum, bronchoalveolar lavage (BAL) or other biological samples) in asthma.

## 5 CONCLUSIONS

In conclusion, the main findings of present meta‐analysis suggested that there was a significant correlation between IL‐33 levels and the severity of asthma. Therefore, IL‐33 levels of either serum or plasma may be regarded as a useful biomarker of asthma or the degree of disease. However, we can not ignore the influences of ethnic and age factors on the IL‐33 levels. It is encouraged that data from multi‐center, well‐designed and large sample size studies should be conducted for validating the clinical value of IL‐33 on asthma.

## AUTHOR CONTRIBUTIONS

**Ranran Zhao**: Conceptualization; data curation; formal analysis; investigation; methodology; software; writing—original draft. **Na Liu**: Data curation; investigation; methodology; software. **Bin Li**: Data curation; investigation; methodology.

## CONFLICT OF INTEREST STATEMENT

The authors declare no conflict of interest.

## Supporting information

Supporting information.

Supporting information.

Supporting information.

Supporting information.

Supporting information.

Supporting information.

Supporting information.

Supporting information.

Supporting information.

Supporting information.

## Data Availability

Data could be obtained from corresponding author.
