# Supplementary material for: Elevated levels of interleukin‐33 are associated with asthma: A meta‐analysis
Source: Immun Inflamm Dis. 2023 Apr 19;11(4):e842. doi: 10.1002/iid3.842 (PMC10116908; doi:10.1002/iid3.842)
Supplement: Supplementary file 2 — Supporting information. [file IID3-11-e842-s003.docx]

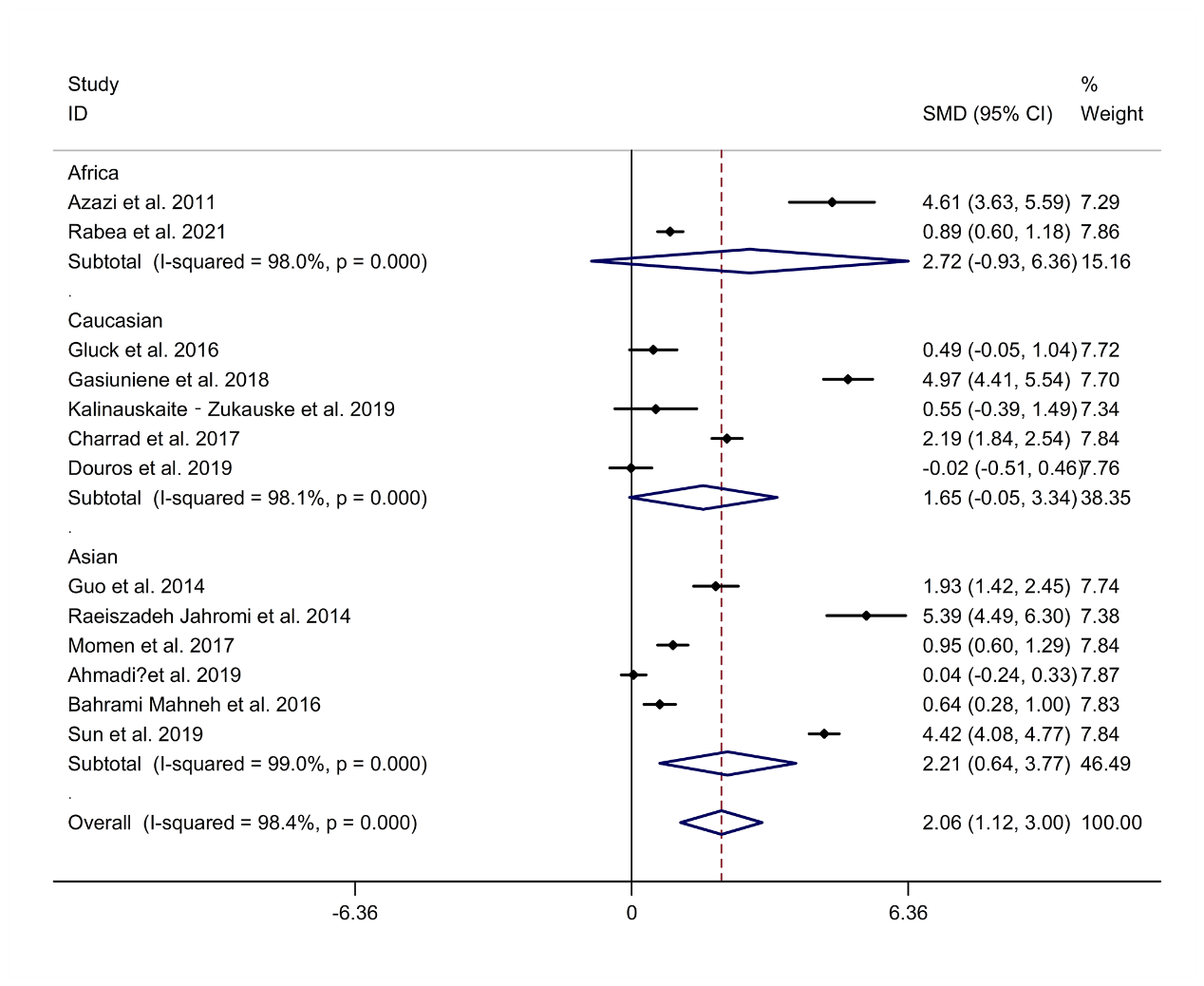


Supplementary figure 1. Subgroup analysis regarding comparison in IL-33 level in serum between asthmatics and HCs in different ethnicities. Abbreviations: CI, confidence interval; HC, healthy control; IL, interleukin; SMD, standard mean difference.
