# Supplementary material for: Elevated levels of interleukin‐33 are associated with asthma: A meta‐analysis
Source: Immun Inflamm Dis. 2023 Apr 19;11(4):e842. doi: 10.1002/iid3.842 (PMC10116908; doi:10.1002/iid3.842)
Supplement: Supplementary file 5 — Supporting information. [file IID3-11-e842-s010.docx]

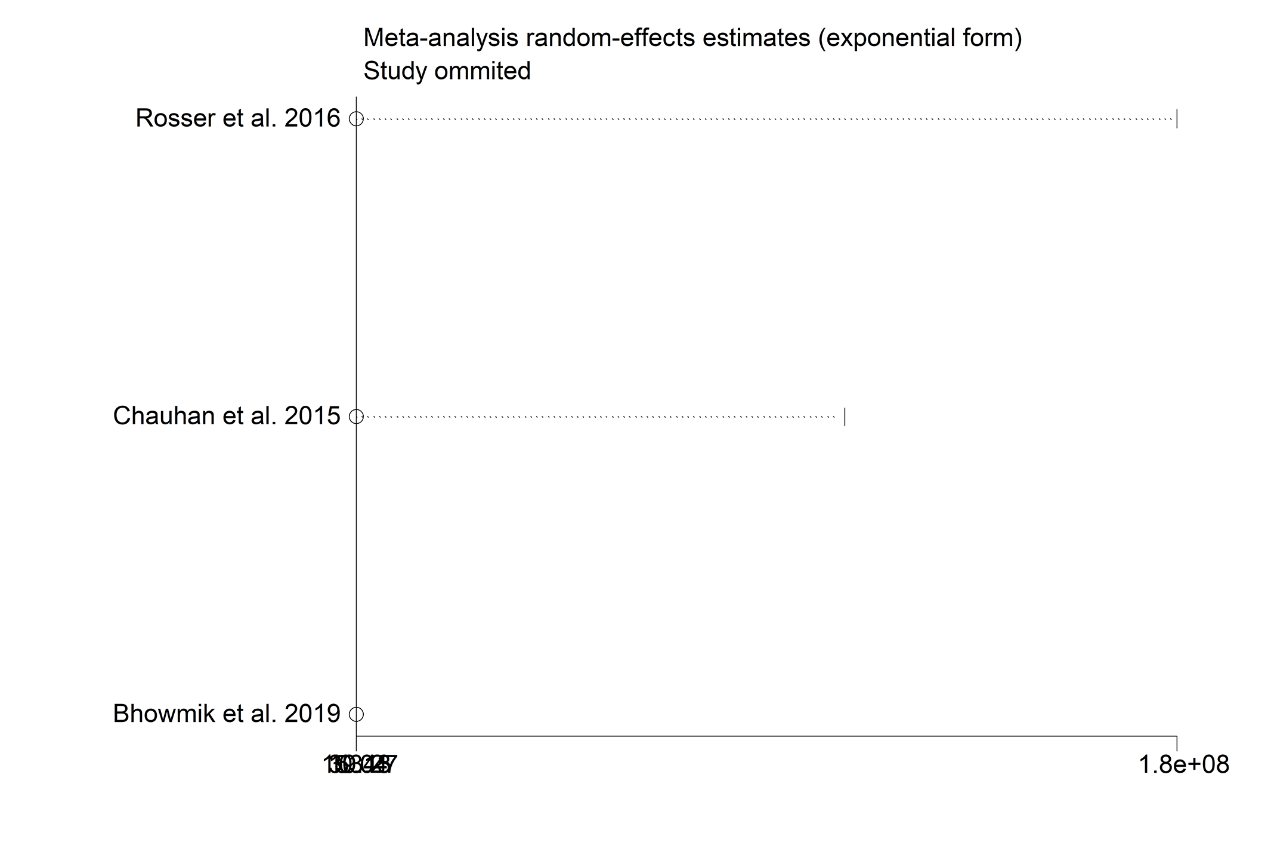


Supplementary figure 4. Sensitivity analyses regarding comparison in IL-33 level in plasma between asthmatics and HCs. Abbreviations: HC, healthy control; IL, interleukin.
