# Supplementary material for: Elevated levels of interleukin‐33 are associated with asthma: A meta‐analysis
Source: Immun Inflamm Dis. 2023 Apr 19;11(4):e842. doi: 10.1002/iid3.842 (PMC10116908; doi:10.1002/iid3.842)
Supplement: Supplementary file 8 — Supporting information. [file IID3-11-e842-s004.docx]

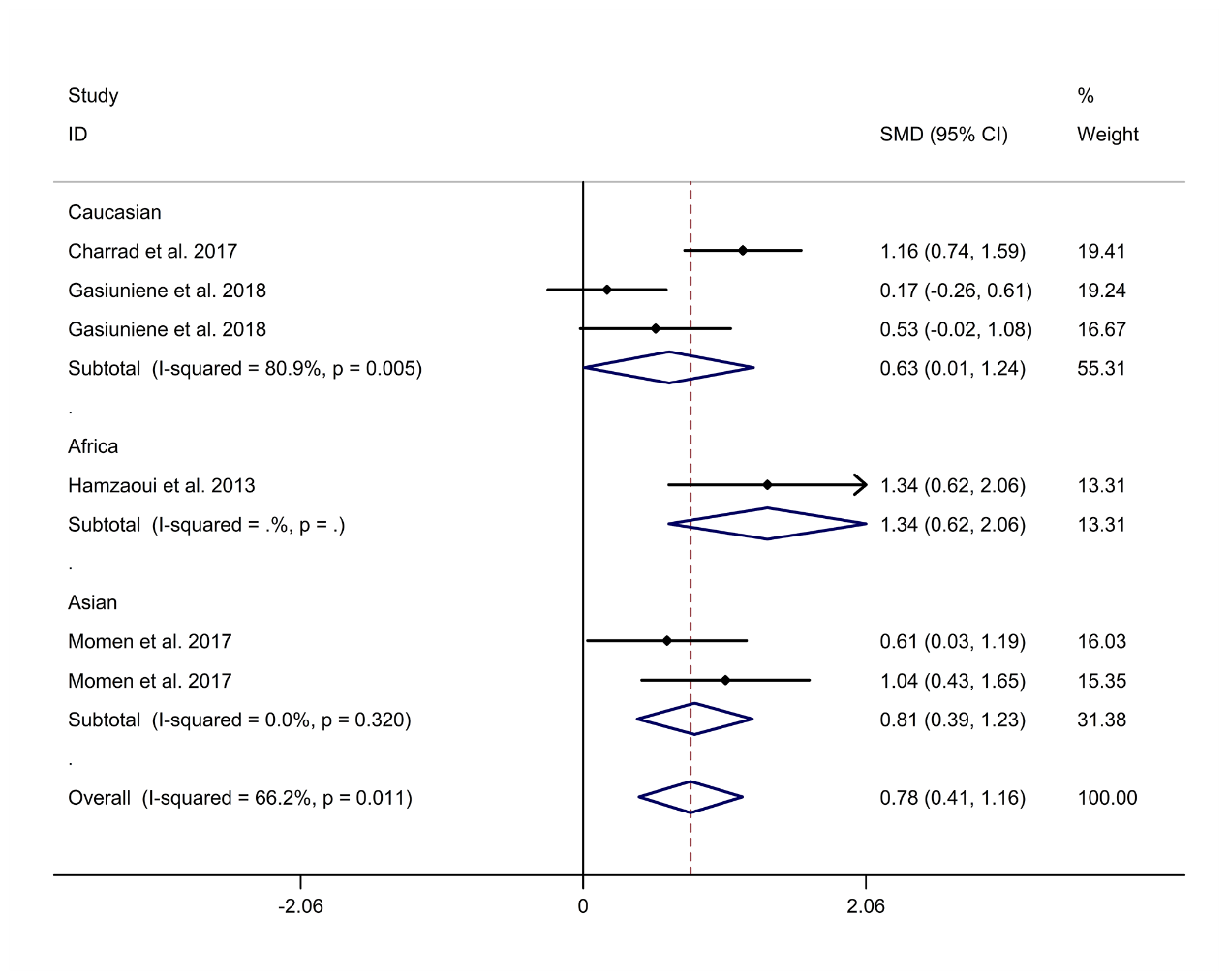


Supplementary figure 7. Subgroup analysis regarding regarding comparison in IL-33 level in serum between moderate/severe asthmatics versus mild asthmatics in different ethnicities. Abbreviations: CI, confidence interval; IL, interleukin; SMD, standard mean difference.
