# Supplementary material for: Elevated levels of interleukin‐33 are associated with asthma: A meta‐analysis
Source: Immun Inflamm Dis. 2023 Apr 19;11(4):e842. doi: 10.1002/iid3.842 (PMC10116908; doi:10.1002/iid3.842)
Supplement: Supplementary file 10 — Supporting information. [file IID3-11-e842-s001.docx]

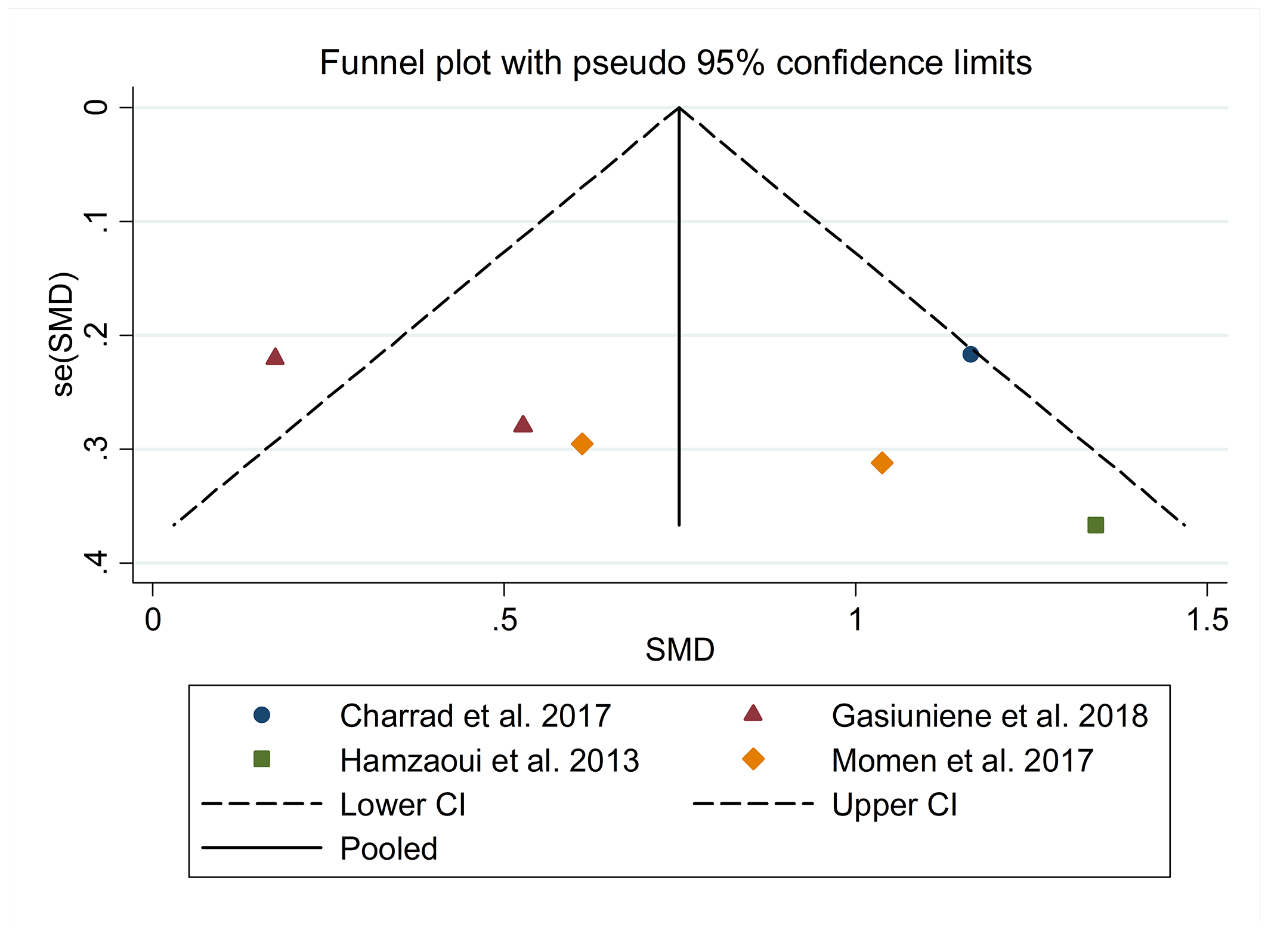


Supplementary figure 9. Funnel plot regarding regarding comparison in IL-33 level in serum between moderate, severe asthmatics and mild asthmatics. Abbreviations: IL, interleukin.
